# Polycystic ovary syndrome and leukocyte telomere length: cross-sectional and longitudinal changes

**DOI:** 10.1530/EJE-22-0462

**Published:** 2022-09-29

**Authors:** Johanna Pölönen, Pekka Pinola, Justiina Ronkainen, Alex I Blakemore, Jessica L Buxton, Juha S Tapanainen, Stephen Franks, Terhi T Piltonen, Sylvain Sebert, Laure Morin-Papunen

**Affiliations:** 1 Department of Obstetrics and Gynecology, University of Oulu and Oulu University Hospital, Medical Research Center, PEDEGO Research Unit, Oulu, Finland; 2 Center for Life Course Health Research, University of Oulu, Oulu, Finland; 3 Department of Life Sciences, College of Health, Medicine and Life Sciences, Brunel University London, London, UK; 4 Department of Metabolism, Digestion and Reproduction, Imperial College London, London, UK; 5 Department of Biomolecular Sciences, School of Life Sciences, Pharmacy and Chemistry, Kingston University London, London, UK; 6 Department of Obstetrics and Gynecology, University of Helsinki and Helsinki University Hospital, Helsinki, Finland; 7 Department of Metabolism, Digestion and Reproduction, Institute of Reproductive and Developmental Biology, Imperial College London, London, UK

## Abstract

**Objective:**

Telomeres are DNA–protein complexes that protect chromosome ends from DNA damage and are surrogate biomarkers of cellular aging. Current evidence, almost entirely from cross-sectional observations, supports negative associations between leukocyte telomere length (LTL) and adverse lifestyle factors and cardiometabolic risk factors. Polycystic ovary syndrome (PCOS), the most common gynecological endocrine disorder, is associated with inflammation and oxidative stress, both factors associated with accelerated telomere attrition. We therefore hypothesized that LTL would be shorter and decrease more rapidly in women with PCOS in comparison to a control population.

**Design:**

This is a population-based cohort study comprising women of Northern Finland Birth Cohort 1966, with clinical examinations at ages 31 and 46. The sample included self-reported PCOS (age 31, *n* = 190; age 46, *n* = 207) and referent women (age 31, *n* = 1054; age 46, *n* = 1324) with data on LTL.

**Methods:**

The association between LTL and PCOS at ages 31 and 46 was analyzed by linear regression models adjusted for BMI, smoking, alcohol consumption and socioeconomic status at the corresponding age.

**Results:**

Women with PCOS had similar mean LTL at ages 31 and 46 (*P > *0.4 for both). The mean LTL change between ages 31 and 46 did not differ between groups (*P* = 0.19). However, we observed a significant LTL attrition between ages 31 and 46 in the reference population (*P* < 0.001), but not in women with PCOS (*P* = 0.96).

**Conclusions:**

This finding may suggest a difference in the LTL attrition rate in women with PCOS, an unexpected finding that might affect their risk of age-related disease. Further research is needed to clarify the underlying mechanisms.

## Introduction

Telomeres are specialized DNA–protein complexes located at the ends of all linear chromosomes (1). Vertebrate telomeres contain thousands of tandem repeats of the sequence TTAGGG, which protect the genome from nucleolytic degradation, thus preserving chromosome integrity and stability (1, 2). At each cell division, a small amount of DNA is lost from every telomere due to the biology of DNA replication: DNA polymerase is incapable of copying the very end of the lagging strand, so the telomeres are not completely replicated. This limitation results in gradual telomere shortening in most somatic cells (3, 4). In germline cells and stem cells, telomere shortening is prevented by the enzyme called telomerase (2). Once telomere length (TL) falls below a critical limit, the cell undergoes senescence and/or apoptosis (1, 5).

Due to its guanine-rich sequence, telomeric DNA is vulnerable to oxidative damage, such as single-strand breaks and erosion, when exposed to free radicals or oxidants (6). Thus, it has been suggested that the rate of telomere shortening also depends on the balance between intracellular oxidative stress and antioxidant defense. Consistent with this hypothesis, shorter telomeres and accelerated telomere shortening have been reported to associate with several chronic syndromes, such as obesity, cardiovascular diseases, glucose metabolism disorders and hyperandrogenism, and further with inflammation and oxidative stress (7, 8, 9, 10, 11, 12, 13).

Polycystic ovary syndrome (PCOS) is the most common endocrine disorder among reproductive-aged women, affecting 6–18% of this population (14). The syndrome is characterized by ovulatory dysfunction, hyperandrogenism and polycystic ovarian morphology (15, 16). The etiology and pathogenesis of PCOS are still not fully understood, but it is thought to be multifaceted, including both genetic and environmental factors where hyperandrogenic exposure in early life seems to be a major culprit (14, 17, 18). Recent genome-wide association studies (GWAS) that have also identified several susceptibility loci for PCOS, however, explain less than 10% of the cases (19, 20). Most women with PCOS are overweight or obese (21, 22), also presenting with increased abdominal fat accumulation compared to controls without obesity (23, 24). Indeed, excess adiposity, insulin resistance and hyperandrogenism are key features of PCOS, promoting chronic inflammation and oxidative stress (25, 26, 27, 28, 29).

Leukocyte telomere length (LTL) has attracted increasing interest as a potential biomarker in the field of reproduction and fertility (13, 30). Infertility has been shown to be associated with shorter TL, suggesting TL predicts both biological and reproductive age (31). Since PCOS is associated with inflammation and oxidative stress, it is hypothesized that the rate of LTL shortening will be faster and LTL may be shorter in PCOS women compared to women that are unaffected (30). To the best of our knowledge, we are lacking longitudinal studies on telomere shortening in women with PCOS and only few cross-sectional studies have been published with conflicting results.

In the present study, we studied a unique, large cohort of women born in 1966 as they turned 46 years. Our aim was two-fold: first, to clarify in a cross-sectional design whether TL differed between women with self-reported PCOS and non-PCOS controls (reference population). The second aim was to perform a longitudinal, follow-up study to determine whether change in LTL between age 31 and pre-menopause differed between PCOS and referent women.

## Subjects and methods

### Data collection and study population

**Figure 1:**
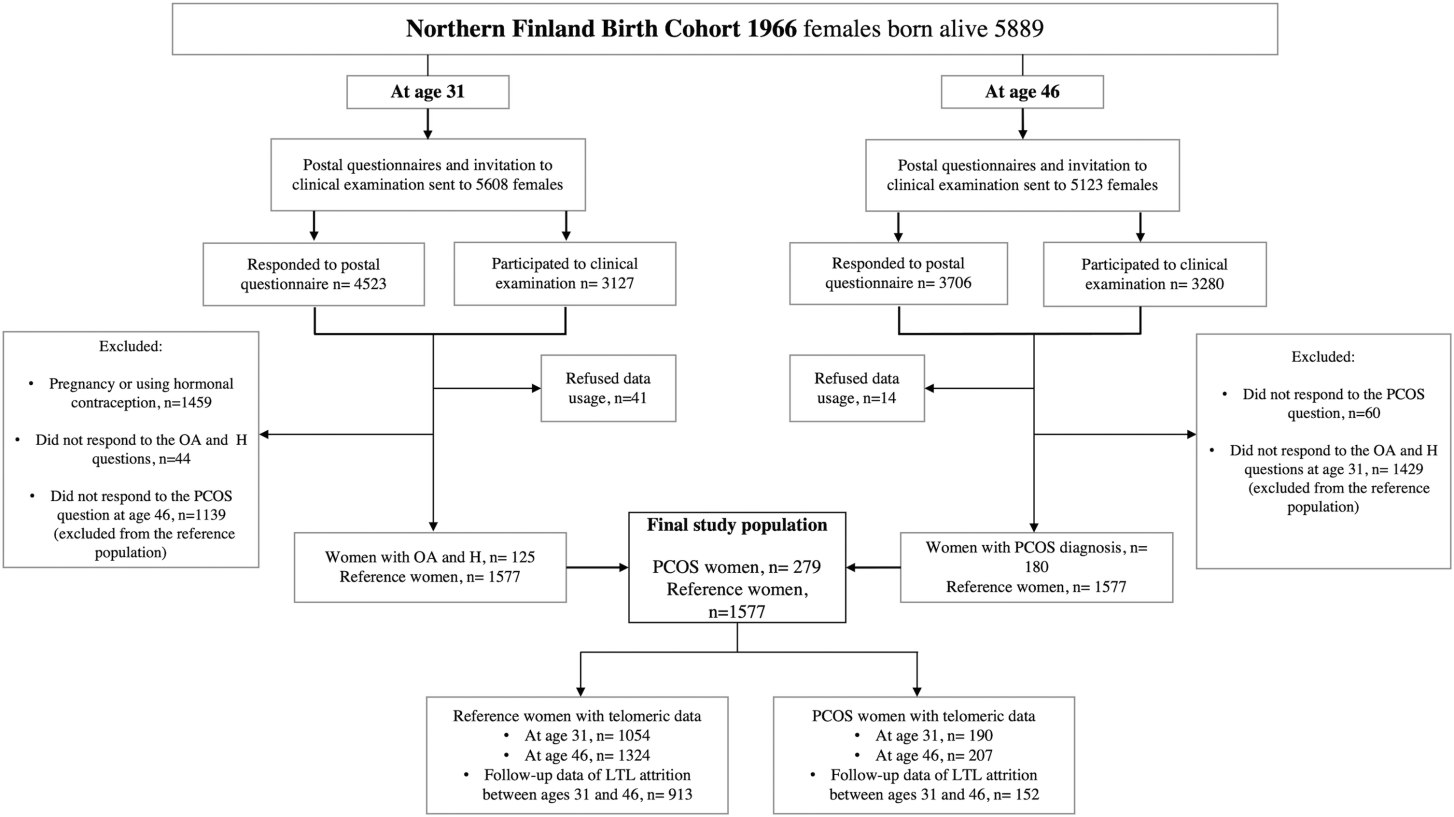
Flow chart of the study. OA, oligo/amenorrhea, H, hirsutism; PCOS, self-reported polycystic ovary syndrome; LTL, leukocyte telomere length.

The study population consisted of the prospective Northern Finland Birth Cohort 1966 (NFBC1966) which comprised all individuals born alive during 1966 (12 231 births, 5889 females, 96.3% of all births during 1966 in that area) (32). At ages 31 and 46, a postal questionnaire and clinical examination were performed (including blood samples, LTL measurement). Detailed cohort profile has been published previously (32) (Fig. 1).

The study followed the principles of the Declaration of Helsinki. The Ethics Committee of the Northern Ostrobothnia Hospital District (EETTMK decision number 94/2011) approved the research on September 17, 2012. All participants took part on a voluntary basis and signed an informed consent to use all the data.

### Definition of PCOS and reference populations

At age 31, the postal questionnaire included questions on oligomenorrhea (‘Is your menstrual cycle often (more than twice a year) longer than 35 days?’) and excessive body hair (‘Do you have troublesome, excessive body hair growth?’). Of the women who responded to these questions, 11.2% (*n* = 330) reported isolated oligo/amenorrhea, 10.9% (*n* = 321) isolated hirsutism and 4.1% (*n* = 125) both oligomenorrhea and hirsutism (OA+H), after excluding pregnant women and those using hormonal preparations (*n* = 1459) or denying data usage (*n* = 41). The postal questionnaire at age 46 included the question: ‘Have you ever been diagnosed as having polycystic ovaries and/or polycystic ovary syndrome (PCOS) during your life?’ to which 180 participants responded ‘yes.’

Consequently, women who reported both OA+H at age 31 and/or diagnosis of PCOS by age 46 were considered as self-reported PCOS cases (*n* = 279). The validity of this questionnaire to distinguish PCOS cases has already been shown in our previous studies from the same cohort, as they presented the typical metabolic, hormonal and psychological profile of PCOS (33, 34, 35, 36). Women without any PCOS symptoms at age 31 and without self-reported diagnosis of PCOS by age 46 were considered as the reference population (*n* = 1577).

The final study population was divided into the following groups: PCOS cases at age 31 (*n* = 190) and at age 46 (*n* = 207), and reference population at age 31 (*n* = 1054) and at age 46 (*n* = 1324). The follow-up data to investigate LTL attrition between ages 31 and 46 included 152 PCOS cases and 913 referent women (Fig. 1).

### Laboratory methods

Genomic DNA was extracted from the blood samples by phenol–chloroform extraction at 31 years and by QIAsymphony DSP DNA Midi Kit (Qiagen) at 46 years. TL was measured from the peripheral blood leukocytes due to sample availability; however, LTL is believed to be a good proxy for TL in other tissues (37, 38). LTL was measured using a monochrome multiplex quantitative PCR method (39). The telomere sequence (T) was quantified relative to a single-copy gene sequence (S), thus providing a T/S ratio for each sample. The T/S ratio is therefore an estimate of the mean relative TL of all telomeres in all leukocytes in the blood DNA sample. T/S values at age 31 were generated using human beta-globin as the single copy reference and the protocol is described in more detail elsewhere (40). T/S at age 46 was assayed in triplicate using albumin as the single copy reference (41, 42), from which the average was used in subsequent analyses. PCR reactions were performed in white 96-well plates on CFX96 Real-time PCR detection system and CFX Manager software (Bio-Rad Laboratories). Samples with coefficient of variation (CV) over 15% between triplicates were run again. Each plate contained an inter-run calibrator, a standard series spanning from 1.2 to 30 ng of genomic DNA and a no-template control. T/S was calculated using 2^−ΔΔCt^ method (43). Mean R^2^ values for standard curves were 0.993 (s.d . = 0.002) and 0.995 (s.d. = 0.002) for telomere and albumin amplicons, respectively. Mean inter-run CV as calculated from calibrator Cq values was 2.77% for telomere and 1.36% for albumin.

Detailed information about laboratory methods for hormonal and metabolic parameters has been published previously (44). At age 31, the concentration of sex hormone-binding globulin (SHBG) was analyzed by fluoroimmunoassay (Wallac, Inc. Ltd., Turku, Finland). At age 46, SHBG was assayed by chemiluminometric immunoassay (Immulite 2000, Siemens Healthcare). The SHBG values from age 31 were transformed to be comparable with the SHBG values analyzed at age 46 using a formula: 0.7615 × old method 31yr SHBG + 0.7088, and the results are reported according to this method (36).

At both ages, the serum levels of total testosterone were measured using Agilent triple quadrupole 6410 LC/MS equipment with an electrospray ionization source operating in positive-ion mode (Agilent Technologies).

Fasting plasma glucose was analyzed by an enzymatic dehydrogenase method (Advia 1800, Siemens Healthcare Diagnostics). Fasting serum insulin was analyzed by a chemiluminometric immunoassay (Advia Centaur XP, Siemens Healthcare Diagnostics). The high-sensitivity C-reactive protein (CRP) was analyzed by an immune-nephelometric assay (BN ProSpec, Siemens Healthcare Diagnostics). The samples were analyzed at NordLab Oulu, a testing laboratory (T113) accredited by the Finnish Accreditation Service (EN ISO 15189).

BMI values at both ages (from clinical examination and postal questionnaire) were combined to create a variable where clinically measured BMI was used if available and self-reported BMI was used in other cases (44).

### Statistical analysis

Since LTL was measured at different times and by using a different reference DNA sample at ages 31 and 46, they were standardized (z-score =(LTL − mean (LTL))/s.d . (LTL)) separately for each measurement.

At both ages, the two independent samples *t*-test was used to compare LTL between the study groups. The association between LTL and PCOS was analyzed by using linear regression models adjusted for Model 1: BMI and Model 2: BMI, smoking, alcohol consumption and socioeconomic status at the corresponding age. Potential effects between PCOS and these adjusting covariates were assessed by using interaction terms between PCOS, BMI, smoking, alcohol consumption and socioeconomic status in linear regression analysis.

Paired samples *t*-test was used to analyze the longitudinal change in LTL between ages 31 and 46 within each study group. The association between LTL attrition rate and PCOS was analyzed by linear regression models adjusted for Model 1: BMI at both ages 31 and 46 and Model 2: BMI, smoking, alcohol consumption and socioeconomic status at both ages 31 and 46.

Two independent samples *t*-test was used to compare the longitudinal change of LTL between ages 31 and 46 between the study groups.

All probability values were two-sided, and *P*-value < 0.05 was considered significant. Analyses were performed with SPSS software, version 25 (SPSS). Benjamini–Hochberg corrections were used to adjust *P*-values for multiple testing.

## Results

### Baseline characteristics of the study population

At both ages, women with PCOS had significantly higher BMI, waist circumferences and serum levels of testosterone, CRP, insulin and fasting glucose and greater insulin resistance than reference women (Table 1).

### Comparison of LTL between PCOS and reference population

Mean LTL did not differ between women with PCOS and the reference population at age 31 (*P* = 0.56) or at age 46 (*P* = 0.41) (Table 1).

**Table 1:** Baseline profile of women with PCOS and reference group at ages 31 and 46. Data are given as means ± s.d. or medians with (25%; 75% quartiles).

Parameters	Women with PCOS		Reference group		*P* value
	*n* (%)	Values	*n* (%)	Values	
Age 31					
LTL (T/S)	190	0.03 ± 0.99	1054	0.07 ± 0.99	0.56
BMI (kg/m^2^)	189	25.06 (17.06; 33.06)	1053	22.85 (17.85; 27.85)	<0.001^a^
Waist (cm)	179	82 (62; 102)	1048	76 (63; 89)	<0.001^a^
Testosterone (nmol/L)	183	1.29 (0.48; 2.10)	1013	0.96 (0.47; 1.46)	<0.001^a^
Fasting glucose (mmol/L)	188	5.17 ± 1.43	1047	4.91 ± 0.51	<0.001^a^
Fasting insulin (mU/L)	187	8.10 (3.1; 13.1)	1045	7.10 (4.25; 9.95)	<0.001^a^
CRP (mg/L)	187	1.06 (−1.85; 3.96)	1037	0.57 (−0.48; 1.63)	<0.001^a^
SHBG (nmol/L)	168	48.65 (8.65; 88.65)	1040	60.70 (23.7; 97.7)	<0.001^a^
HOMA–IR	180	1.02 (0.45; 1.60)	1012	0.92 (0.54; 1.29)	<0.001^a^
Alcohol consumption (g/day)	188	1.6 (−3.6; 6.8)	1033	2.20 (−2.9; 7.3)	0.36
Smoking	189		1050		0.14
Non-smoker	90 (47.6%)		528 (50.3%)		
Former smoker	43 (22.8%)		279 (26.6%)		
Current smoker	56 (29.6%)		243 (23.1%)		
Socioeconomic status	190		1051		0.46
Farmers	7 (3.7%)		29 (2.8%)		
Entrepreneurs and higher officer	32 (16.8%)		225 (21.4%)		
Lower officers	81 (42.6%)		466 (44.3%)		
Blue collars	31 (16.3%)		148 (14.1%)		
Students, pensioners, long-term unemployed and other unclassified	39 (20.5%)		183 (17.4%)		
Age 46					
LTL (T/S)	207	−0.05 ± 0.95	1324	0.01 ± 1.02	0.41
BMI (kg/m^2^)	207	27.20 (20.2; 34.2)	1324	25.24 (19.24; 31.24)	<0.001^a^
Waist (cm)	205	88.50 (70.5; 106.5)	1316	84.00 (67; 101)	<0.001^a^
Testosterone (nmol/L)	206	0.89 (0.45; 1.32)	1321	0.82 (0.40; 1.25)	0.02^a^
Fasting glucose (mmol/L)	205	5.53 ± 1.39	1305	5.32 ± 0.57	<0.001^a^
Fasting insulin (mU/L)	204	8.40 (2.13; 14.68)	1312	7.10 (1.5; 12.7)	<0.001^a^
CRP (mg/L)	206	0.85 (−0.72; 2.43)	1320	0.69 (-0.54; 1.93)	0.12
SHBG (nmol/L)	206	49.30 (16.85; 81.75)	1321	54.30 (17.3; 91.3)	0.01^a^
HOMA–IR	203	2.00 (0.42; 3.59)	1303	1.66 (0.27; 3.10)	<0.001^a^
Alcohol consumption (g/day)	202	2.40 (−5.20; 10.0)	1323	2.90 (−4.60; 10.4)	0.46
Smoking	200		1307		0.45
Non-smoker	115 (57.5%)		762 (58.3%)		
Former smoker	44 (22.0%)		321 (24.6%)		
Current smoker	41 (20.5%)		224 (17.1%)		
Socioeconomic status	199		1295		0.21
Farmers	4 (2.0%)		12 (0.9%)		
Entrepreneurs and higher officer	48 (24.1%)		325 (25.1%)		
Lower officers	44 (22.1%)		283 (21.9%)		
Blue collars	84 (42.2%)		597 (46.1%)		
Students, pensioners, long-term unemployed and other unclassified	19 (9.5%)		78 (6.0%)		

The differences between women with PCOS and the reference population were analyzed by Student’s *t*-test or Mann–Whitney *U* test when appropriate. Categorical variables were analyzed by Pearson’s Chi-squared test. *P*-values < 0.05 were considered as significant.

CRP, C-reactive protein, HOMA–IR, homoeostasis model assessment–insulin resistance; PCOS, self-reported polycystic ovary syndrome; SHBG, sex hormone-binding globulin; T/S, single copy gene ratio.

^a^*P*-values were significant after Benjamini–Hochberg corrections.

LTL was not associated with PCOS at age 31 (*P* = 0.59) (Table 2) and the results did not change after adjustments (Model 1: *P* = 0.87, Model 2: *P* = 0.22). Similarly, there was no statistically significant association between LTL and PCOS at age 46 (*P* = 0.41) and the results did not change after adjustments (Model 1: *P* = 0.48, Model 2: *P* = 0.71) (Table 2). The levels of serum testosterone were not significantly correlated with LTL in either of the study groups (Age 31: PCOS women: r = 0.033, *P* = 0.66; reference population: r = 0.030, *P* = 0.35. Age 46: PCOS women: r = −0.108, *P* = 0.12; reference population: r = 0.008, *P* = 0.78).

**Table 2:** Association between LTL and PCOS. The B, 95% CI of B and P values were estimated for the association between LTL and PCOS by using linear regression models. *P*-value < 0.05 was considered significant.

	Beta (95% CI)	*P* value
Age = 31 years		
Crude	−0.042 (−0.194; 0.111)	0.59
Model 1	−0.013 (−0.168; 0.143)	0.87
Model 2^a^	−0.114 (−0.294; 0.067)	0.22
Age = 46 years		
Crude	−0.062 (−0.209; 0.086)	0.41
Model 1	−0.053 (−0.202; 0.096)	0.48
Model 2^b^	−0.029 (−0.182; 0.124)	0.71

Crude, no adjustments. Model 1, adjustment for BMI. Model 2, adjustment for BMI, smoking, alcohol consumption and socioeconomic status.

LTL, leukocyte telomere length; PCOS, self-reported polycystic ovary syndrome.

^a^The interaction term between PCOS and alcohol consumption was included into the model.

^b^The interaction terms between alcohol consumption and smoking & alcohol consumption and socioeconomic status were included into the model.

### Rates of age-related shortening of LTL

**Figure 2:**
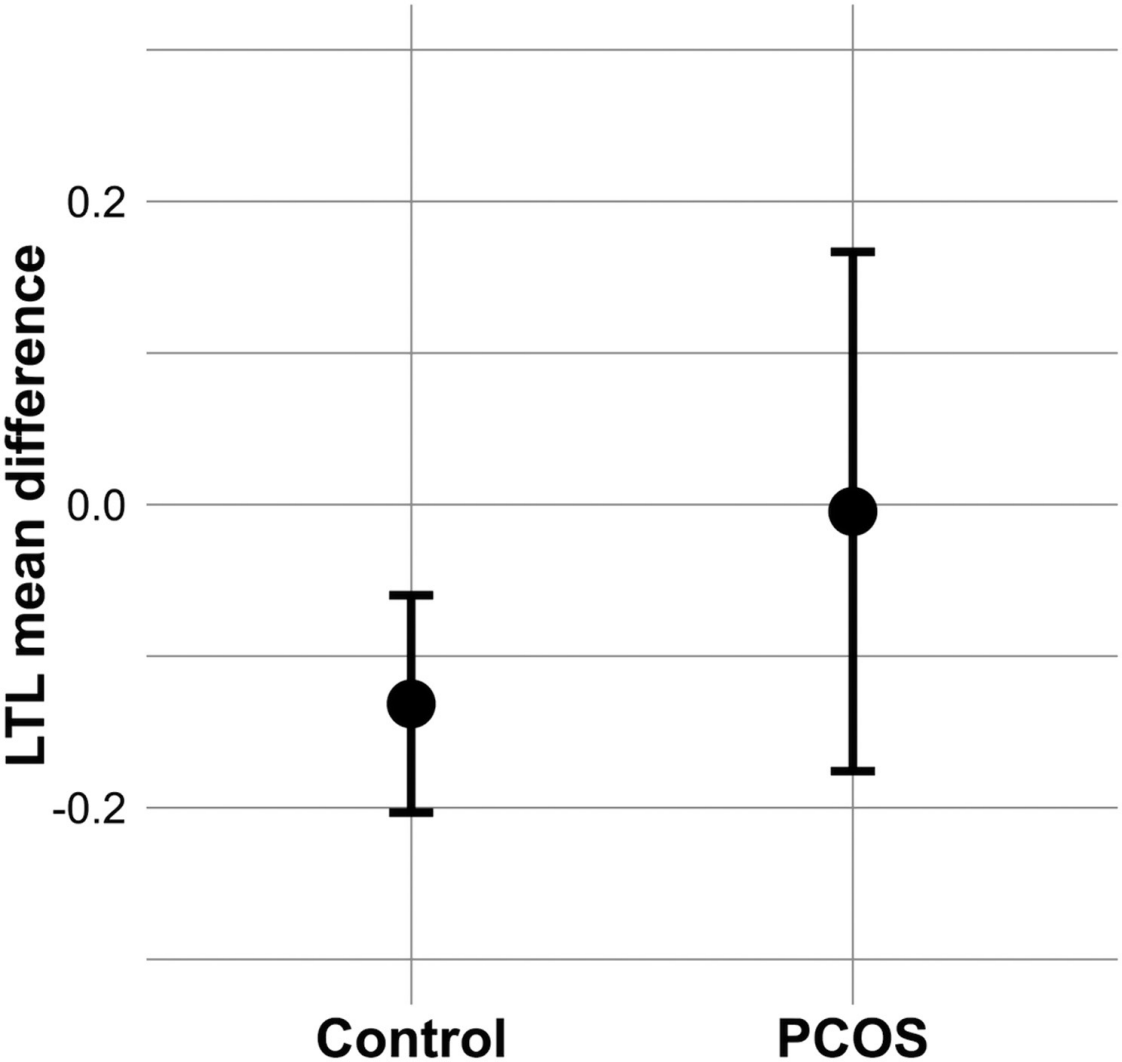
Mean difference of LTL z-scores between ages 31 and 46 with 95% CIs in PCOS and reference population. LTL, leukocyte telomere length; PCOS, self-reported polycystic ovary syndrome.

Since telomeres shorten with age, we explored the degree of change in LTL over the 15 years study period, in affected and unaffected women. We observed a significant negative mean difference (MD) of LTL z-scores between ages 31 and 46 in the reference population (MD: −0.13 ± 1.10, 95% CI = −0.20; (−0.060), *P* < 0.001) (Fig. 2), whereas there was no significant difference in the MD of LTL z-scores between ages 31 and 46 in the PCOS group (MD: −0.0045 ± 1.10, 95% CI = −0.18; 0.17, *P* = 0.96) (Fig. 2). However, the MD of LTL z-scores between the ages of 31 and 46 did not differ in a cross-comparison between the PCOS women and the reference population (MD: −0.0045 ± 1.10 vs MD: −0.13 ± 1.10, 95% CI = −0.30; 0.060, *P* = 0.19).

Moreover, in the linear regression analysis, PCOS was not significantly associated with LTL z-score difference (*P* = 0.19) (Table 3). The results did not change after adjustments.

**Table 3:** Association between PCOS and changes in LTL between ages 31 and 46. The B, 95% CI of B and P values were estimated for the association between PCOS and LTL between ages 31 and 46 by using linear regression models. *P*-value < 0.05 was considered significant.

	Beta (95% CI)	*P* value
Crude	0.127 (−0.062; 0.316)	0.19
Model 1	0.103 (−0.088; 0.295)	0.29
Model 2^a^	0.134 (−0.062; 0.330)	0.18

Crude, no adjustments. Model 1, adjustment for BMI at age 31 and 46. Model 2, adjustment for BMI, smoking, alcohol consumption and socioeconomic status at ages 31 and 46.

LTL, leukocyte telomere length; PCOS, self-reported polycystic ovary syndrome.

^a^The interaction terms between alcohol consumption at ages 31 and 46 & smoking at age 46 and alcohol consumption at age 46 were included into the model.

## Discussion

The mean LTL did not differ significantly between women with PCOS and the reference population either at the age of 31 or 46, despite the unfavorable metabolic profile observed in women with PCOS. No statistically significant association between LTL and PCOS was found at the ages of 31 or 46. The results did not change after adjustments. In contrast to our original hypothesis, the mean change in LTL z-scores between ages 31 and 46 was not significantly different in the PCOS group from the reference population, despite demonstrating the expected age-related, statistically significant decline in the reference population.

To our knowledge, this is the first longitudinal population-based study prospectively investigating the changes in LTL over time in women with PCOS compared to non-PCOS reference population. Interestingly, the MD of LTL between ages 31 and 46 years was negative in the reference population, but not in the PCOS group suggesting a slower telomere shortening rate in PCOS women. This observation does not support our original hypothesis, as previous studies by our group and others have established that PCOS women are at risk for adverse cardiometabolic disorders (obesity, insulin resistance, pro-inflammation) (23, 27, 24, 44, 45) that are often associated with shorter LTL among the general population (6, 8, 10, 30, 46). In line with these findings, a previous large GWAS on LTL in UK biobank showed that shorter LTL is causally linked with coronary artery disease and lower life expectancy (47).

While replication in additional cohorts is needed, there are some interesting possible explanations for our findings. In some studies, PCOS women have been reported to experience menopause later than the reference population (48, 49). Reproductive performance has been suggested to be a good marker for a woman’s general health later in life, and an extended reproductive lifespan has been correlated with long-term health and longevity, probably caused, at least partly, by longer estrogen exposure (50, 51). Longer exposure to endogenous estrogen or use of postmenopausal hormone therapy has been associated with greater TL and a lower risk of age-related diseases (50, 52). In keeping with this hypothesis, women with PCOS, despite numerous metabolic risk factors, do not seem to experience as many cardiometabolic events as might be expected (49, 53, 54), although opposite results have also been reported (44, 45, 55). These findings raise interesting questions about the relationship between estrogen levels and TLs. Estrogen levels were not routinely measured in our study population, and we were therefore not able to evaluate this association. The potential mechanisms underlying our findings remain therefore to be clarified and the complex interactions between PCOS, estrogen levels and LTL need more investigation.

The present study did not support evidence for a difference in LTL between women with PCOS and women in the reference group. Until now, there have been only few comparative studies exploring the association between TL and PCOS. The first Chinese study showed shorter LTL in women with PCOS (*n* = 698) compared to the control group (*n* = 611) after adjusting for age (30). Conversely, a Brazilian study, comparing 150 women with PCOS to 124 healthy women, did not find any significant difference in LTL between the 2 groups, but socioeconomic status or ethnicity was not taken into account and the age of the study population varied from 13 to 45 years (13). In a more recent study focusing on the TL in cumulus cells of immature and mature oocytes, TLs did not differ between women with PCOS and controls (56). However, the same research group found reduced TLs in the leukocytes in the PCOS group compared to controls (56). Further, a Chinese study reported increased TLs in granulosa cells but not in the leukocytes in women with PCOS compared to controls (57). Conversely, another Chinese study of 40 women with PCOS and 35 control women found that affected women had significantly longer LTL, after adjusting for age (58). The authors hypothesized that androgens may promote telomerase activity and thus be linked to the increased TL in PCOS women. Supporting this, an Argentinian study showed in a retrospective, cross-sectional setting conducted on young women that the PCOS women had longer TL compared with controls and that higher testosterone levels correlated positively with TL (59). In the present study, however, we did not find any significant correlation between serum testosterone levels and LTL. These findings are surprising, as the PCOS hyperandrogenic phenotypes have been associated with the most unbeneficial metabolic phenotypes in PCOS (60, 61), and we would have expected shorter LTL in this group of women. The relationship between androgen levels and LTL therefore remains to be elucidated in further studies.

The discrepancies between these findings might result from differences between the study populations, as all the abovementioned studies are case-control reports with participants recruited randomly irrespective of age, social status, genetic background and of completely different ethnic groups (13, 30, 32, 57, 58). Discrepancies may also stem from the definition of PCOS and reference populations in each study. Lastly, the small number of participants in some studies could reduce statistical power. Environmental factors contribute to the risk of PCOS and differences in these factors between the study populations can also lead to contradictory results in telomere outcomes. In this respect, we think that our results are robust due to the large sample size, population-based study with two consecutive LTL measures and possibility to control for some confounding factors and risks of bias.

The main strength of our study is the prospective, population-based follow-up design. To our knowledge, this is the first study to examine LTL change over time in women with PCOS. Birth cohort follow-up studies are more suitable for investigating the role of telomere shortening in PCOS than cross-sectional studies, as they offer a methodologically more suitable approach, for example for follow-up at different stages of life (62). The NFBC1966 study offers a unique opportunity to investigate telomere shortening in women with PCOS and to make comparisons with a reference group. The study population is also remarkably homogeneous with respect to its genetic structure, due to the isolation, founder effect and multiple bottlenecks of the Finnish population (63, 64) and the NFBC1966 consists almost entirely of Caucasians. Most importantly, it included only women of the same age, which is highly beneficial for telomere studies, as data do not have to be corrected for age, a source of potential bias.

This study also has some limitations. The total number of women with missing telomere data did not differ between the study groups at age 31 (PCOS: *n*  = 89 (31.9%) vs reference group: *n*  = 523 (33.2%), *P* = 0.37) but was significantly greater in the PCOS group at age 46 (PCOS: *n*  = 72 (25.8%) vs reference population: *n*  = 253 (16.0%), *P* < 0.001). This feature could have decreased the significance of the analyses in the PCOS group. The symptoms and diagnosis of PCOS were self-reported. Consequently, our study population probably involved women with milder hormonal and metabolic disorders in comparison to women diagnosed with PCOS attending infertility clinics for example. This study feature may have decreased the significance of the association between PCOS and LTL and prevented us from generalizing to all phenotypes of PCOS. We acknowledge that hirsutism might be over-reported and ovarian ultrasonography was not available to aid the diagnosis of PCOS. However, this definition is consistent with the National Institutes of Health, the Rotterdam and the New Guidelines criteria for diagnosis of PCOS (15, 16). Moreover, in this same study population, we have previously shown that self-reported PCOS can identify women with the typical endocrine, metabolic and psychological profiles of PCOS (34, 35, 36), and this was also observed in the present study. DNA extraction as well as LTL measurement methods were slightly different between 31-year and 46-year follow-ups, so we were not able to monitor the absolute change in LTL over time. To overcome this, we used standardized LTL values, where the rate of the change may be observed rather than the absolute change. The higher prevalence of obesity in PCOS women as well as differences in weight gain through life between the two study groups could have biased the results, as both parameters have been associated with a faster attrition rate in TL (9, 65). However, LTL did not differ between PCOS and controls in the crude models and adjustments for BMI did not change the results. We have also previously shown in this same population that weight gain was similar between ages 31 and 46 when comparing controls and PCOS women (24). Another important limitation is also that the study population was relatively young to expect significant telomere shortening. We do acknowledge that a larger time frame may have offered more opportunity to observe differences in LTL, although there are too little comparable data available on the optimal follow-up length. The follow-ups were decided to prospectively study transition periods in adulthood and, unfortunately, biological samples and PCOS-related questionnaires were only available at ages 31 and 46.

This study made two important observations: First, it does not support evidence for a risk of shorter LTL in women with PCOS. Secondly, the 15-year shortening rate of LTL appeared not to be faster in women with PCOS compared to the rest of the population. These results were observed despite the presence of a less favorable metabolic profile in women with PCOS. This was an unexpected finding that might affect their risk of age-related disease. However, because of the aforementioned limitations of our study and before concluding whether there is a causal association between PCOS and LTL or not, further research is needed to clarify the underlying mechanisms and compare these associations with other biomarkers of biological aging.

## Declaration of interest

Terhi T Piltonen is on the editorial board of the *European Journal of Endocrinology*. Terhi T Piltonen was not involved in the review or editorial process for this paper, on which he/she is listed as an author. The other authors declare that they do not have any conflict of interest.

## Funding

The follow up of the NFBC1966 study received financial support from University of Oulu Grant no. 65354, 24000692; Oulu University Hospital Grant no. 2/97, 8/97, 24301140; Ministry of Health and Social Affairs Grant no. 23/251/97, 160/97, 190/97; National Institute for Health and Welfare, Helsinki Grant no. 54121; Regional Institute of Occupational Health, Oulu, Finland Grant no. 50621, 54231; ERDF European Regional Development Fund Grant no. 539/2010 A31592. S S, M R J, A B and J R acknowledge funding from the European Union’s Horizon 2020 research and innovation programme under grant agreement n°633595, DynaHEALTH; S S, M R J, J R received additional support from the following: H2020-733206 LifeCycle, H2020-824989 EUCANCONNECT, H2020-873749 LongITools, and the JPI HDHL, PREcisE project, ZonMw the Netherlands no. P75416. T P acknowledges funding from the Academy of Finland under grant number 315921and 321763.

## Author contribution statement

J P: study design, execution, analysis, manuscript drafting and critical discussion. P P: study design, execution, analysis, manuscript drafting and critical discussion. J R: study design, analysis, manuscript drafting and critical discussion. A I B: manuscript drafting and critical discussion. J L B: manuscript drafting and critical discussion. J S T: manuscript drafting and critical discussion. S F: manuscript drafting and critical discussion. T P: manuscript drafting and critical discussion. S S: study design, analysis, manuscript drafting and critical discussion. L M-P: study design, execution, manuscript drafting and critical discussion.
